# T Cell‐Derived Apoptotic Extracellular Vesicles Hydrolyze cGAMP to Alleviate Radiation Enteritis via Surface Enzyme ENPP1

**DOI:** 10.1002/advs.202401634

**Published:** 2024-06-18

**Authors:** Yang Zhou, Lili Bao, Shengkai Gong, Geng Dou, Zihan Li, Zhengyan Wang, Lu Yu, Feng Ding, Huan Liu, Xiayun Li, Siying Liu, Xiaoshan Yang, Shiyu Liu

**Affiliations:** ^1^ College of Life Sciences Northwest University Xi'an Shaanxi 710069 China; ^2^ State Key Laboratory of Oral & Maxillofacial Reconstruction and Regeneration National Clinical Research Center for Oral Diseases Shaanxi International Joint Research Center for Oral Diseases Center for Tissue Engineering School of Stomatology The Fourth Military Medical University Xi'an Shaanxi 710032 China; ^3^ Department of Orthodontics School and Hospital of Stomatology Cheeloo College of Medicine Shandong University & Shandong Key Laboratory of Oral Tissue Regeneration & Shandong Engineering Research Center of Dental Materials and Oral Tissue Regeneration & Shandong Provincial Clinical Research Center for Oral Diseases Jinan Shandong 250012 China; ^4^ Department of Periodontology School and Hospital of Stomatology Cheeloo College of Medicine Shandong University & Shandong Key Laboratory of Oral Tissue Regeneration & Shandong Engineering Research Center of Dental Materials and Oral Tissue Regeneration & Shandong Provincial Clinical Research Center for Oral Diseases Jinan Shandong 250012 China; ^5^ State Key Laboratory of Oral & Maxillofacial Reconstruction and Regeneration National Clinical Research Center for Oral Diseases Shaanxi International Joint Research Center for Oral Diseases Department of Radiology School of Stomatology The Fourth Military Medical University Xi'an Shaanxi 710032 China; ^6^ Department of Otolaryngology Head and Neck Surgery Peking University Third Hospital Beijing 100871 China; ^7^ State Key Laboratory of Oral & Maxillofacial Reconstruction and Regeneration National Clinical Research Center for Oral Diseases Shaanxi Clinical Research Center for Oral Diseases Department of Orthodontics School of Stomatology The Fourth Military Medical University Xi'an Shaanxi 710032 China; ^8^ Stomatology Hospital School of Stomatology Southern Medical University Guangzhou Guangdong 510280 China

**Keywords:** apoptotic extracellular vesicles, cGAMP, cGAS‐STING pathway, ENPP1, radiation enteritis

## Abstract

Radiation enteritis is the most common complication of pelvic radiotherapy, but there is no effective prevention or treatment drug. Apoptotic T cells and their products play an important role in regulating inflammation and maintaining physiological immune homeostasis. Here it is shown that systemically infused T cell‐derived apoptotic extracellular vesicles (ApoEVs) can target mice irradiated intestines and alleviate radiation enteritis. Mechanistically, radiation elevates the synthesis of intestinal 2′3′ cyclic GMP‐AMP (cGAMP) and activates cyclic GMP‐AMP synthase (cGAS)‐stimulator of interferon genes (STING) proinflammatory pathway. After systemic infusion of ApoEVs, the ectonucleotide pyrophosphatase phosphodiesterase 1 (ENPP1) enriches on the surface of ApoEVs hydrolyze extracellular cGAMP, resulting in inhibition of the cGAS‐STING pathway activated by irradiation. Furthermore, after ApoEVs are phagocytosed by phagocytes, ENPP1 on ApoEVs hydrolyzed intracellular cGAMP, which serves as an intracellular cGAMP hydrolyzation mode, thereby alleviating radiation enteritis. The findings shed light on the intracellular and extracellular hydrolysis capacity of ApoEVs and their role in inflammation regulation.

## Graphical abstract

Liu and colleagues find that T cell‐derived apoptotic extracellular vesicles (ApoEVs) alleviate radiation enteritis. They reveal that ENPP1 enriched on the ApoEVs surface hydrolyzes intracellular and extracellular cGAMP, thereby inhibiting the cGAS‐STING pathway activated by irradiation, promoting inflammation resolution and alleviating radiation enteritis. Their study reveals the intracellular and extracellular hydrolysis capacity of ApoEVs.

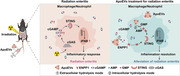

## 1 Introduction

As an important digestive organ, the intestine is responsible for absorbing nutrients from food and participates in immune responses. Simultaneously, as a radiation‐sensitive organ, the intestine is vulnerable to injury during abdominal or pelvic radiotherapy, resulting in radiation enteritis with loss of intestinal crypt, diarrhea, and even death.^[^ ^1^ ^]^ In patients with abdominal or pelvic radiotherapy, the incidence of acute intestinal toxicity symptoms is 60–80% and continues to grow.^[^ ^1^ ^]^ Therefore, therapeutic strategies for radiation enteritis are of great importance.

Intestinal immune imbalance is an alarm for the development of radiation enteritis.^[^ ^2^ ^]^ Ionizing radiation (IR) triggers an inflammatory response, which is due to the increased synthesis of reactive oxygen species, reactive nitrogen species, pro‐inflammatory cytokines, and chemokines after irradiation, leading to the recruitment and activation of multiple immune cells.^[^ ^2b^ ^]^ Moreover, irradiation causes cells to release DNA and RNA into the cytoplasm, and these molecules can be sensed by immune cells via a multitude of nucleic acid‐recognition pathways.^[^ ^3^ ^]^ Of these, the occurrence of DNA in the cytosol constitutes a potent trigger for the innate immune system.^[^ ^2b^ ^]^ In response to double‐stranded DNA in the cytosol, the enzyme cyclic GMP‐AMP synthetase (cGAS) promotes 2′3′ cyclic GMP‐AMP (cGAMP) synthesis.^[^ ^4^ ^]^ As a second messenger molecule, cGAMP binds and activates the endoplasmic reticulum surface receptor stimulator of interferon genes (STING). The activated STING recruits TANK binding kinase 1 (TBK1) and then phosphorylates interferon regulatory factor 3 (IRF3),^[^ ^5^ ^]^ leading to the production of key inflammatory cytokines involved in innate immunity, such as type I interferon (IFN‐I).^[^ ^4^, ^6^ ^]^ It has been shown that excessive activation of the cGAS‐STING pathway disrupts intestinal homeostasis. Intestinal cGAS is upregulated during human and murine colitis,^[^ ^7^ ^]^ and inhibition of cGAS activity alleviates colitis.^[^ ^8^ ^]^ In addition, increased STING expression is a feature of inflammatory bowel disease.^[^ ^9^ ^]^ In the presence of STING agonists, colitis is greatly exacerbated in mice treated with dextran sulfate sodium.^[^ ^10^ ^]^ Moreover, after total body irradiation, cGAS‐STING‐dependent IFN‐I in the intestine gradually increases, leading to acute inflammation.^[^ ^11^ ^]^ Therefore, limiting excessive activation of the cGAS‐STING pathway could be essential to maintain intestinal homeostasis and improve radiation enteritis.

Lymphocyte apoptosis plays an important role in regulating inflammation and maintaining physiological homeostasis.^[^ ^12^ ^]^ The uptake of apoptotic T cells by macrophages induces the release of TGF‐β, leading to upregulation of Tregs.^[^ ^13^ ^]^ It has also been proven that apoptotic T cells directly secrete TGF‐β to inhibit the production of pro‐inflammatory cytokine in macrophages.^[^ ^14^ ^]^ During apoptosis, in addition to releasing cytokines, cells also release a large number of apoptotic extracellular vesicles (ApoEVs),^[^ ^15^ ^]^ which have strong immunosuppressive potential.^[^ ^16^ ^]^ However, whether ApoEVs released by T cells could alleviate radiation‐induced inflammation remains to be elucidated.

In this study, we first found that ApoEVs released by T cells could target the intestines of abdominal irradiated mice and alleviate radiation enteritis. Irradiation enhanced the synthesis of cGAMP and activated the cGAS‐STING pathway in the intestine, which was inhibited through ApoEVs treatment. Subsequently, we revealed that the surface of ApoEVs was enriched with ectonucleotide pyrophosphatase phosphodiesterase 1 (ENPP1), which was the only detectable cGAMP hydrolase reported in mammals.^[^ ^17^ ^]^ The ENPP1 on ApoEVs hydrolyzed extracellular and intracellular cGAMP to inhibit the activation of the cGAS‐STING pathway, thus alleviating radiation enteritis. These results revealed the hydrolyzing ability of ApoEVs and their role in inflammation regulation.

## 2 Results

### 2.1 Isolation and Characterization of T Cell‐Derived Apoptotic Extracellular Vesicles (ApoEVs)

**Figure 1:**
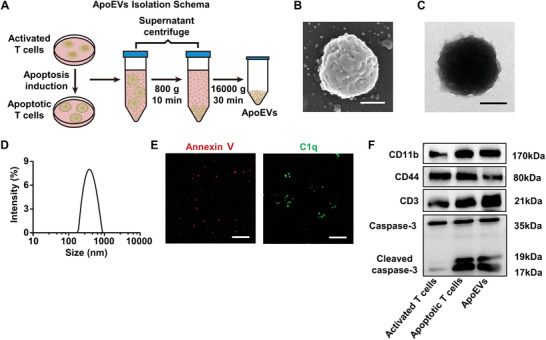
Isolation and characterization of T cell‐derived apoptotic extracellular vesicles (ApoEVs). A) Schematic diagram of the ApoEVs isolation. Murine‐activated T cells were induced for apoptosis with 500 nm STS for 12 h and then the apoptotic cell suspension was isolated using a differential centrifuge system to get ApoEVs. B) Representative scanning electron microscopy (SEM) image of ApoEVs. Scale bar, 200 nm. C) Representative transmission electron microscopy (TEM) image of ApoEVs. Scale bar, 200 nm. D) Size distribution of ApoEVs measured by dynamic light scattering (DLS). E) Annexin V and C1q staining of ApoEVs. Scale bar, 20 µm. F) Protein characterization of activated T cells, apoptotic T cells, and ApoEVs by Western blot.

To study apoptotic vesicles released by apoptotic T cells, we first extracted T cells from mice. The extracted splenic T cells were activated by CD3ε and CD28 antibodies. Activated T cells showed larger size than naive T cells (Figure S1A, Supporting Information) and showed higher CD25 expression levels (Figure S1B, Supporting Information). Staurosporine (STS) was used to induce apoptosis of activated T cells.^[^ ^18^ ^]^ Then ApoEVs were isolated by differential centrifugation (**Figure** **1**A).^[^ ^19^ ^]^ Scanning electron microscopy (SEM) and transmission electron microscopy (TEM) analysis showed that ApoEVs were spherically shaped (Figure 1B,C). As assessed by dynamic light scattering (DLS), the size of ApoEVs varied within the 150 to 900 nm range (Figure 1D), which was consistent with SEM and TEM images (Figure 1B,C). Next, immunofluorescent staining showed that ApoEVs were positive for apoptosis‐specific markers phosphatidylserine (PtdSer, indicated by Annexin V staining) and complement component 1q (C1q) (Figure 1E). Western blot confirmed that activated T cells, apoptotic T cells, and ApoEVs expressed the same membrane markers, including CD11b, CD44, and CD3 (Figure 1F). In addition, Cleaved caspase‐3, a specific marker of apoptotic cells, was detectable in apoptotic T cells and ApoEVs (Figure 1F), indicating the successful induction of apoptosis in T cells. Altogether, these data suggested that the ApoEVs used in this study conformed to the specific characteristics of ApoEVs.

### 2.2 ApoEVs Administration Alleviates Radiation Enteritis

**Figure 2:**
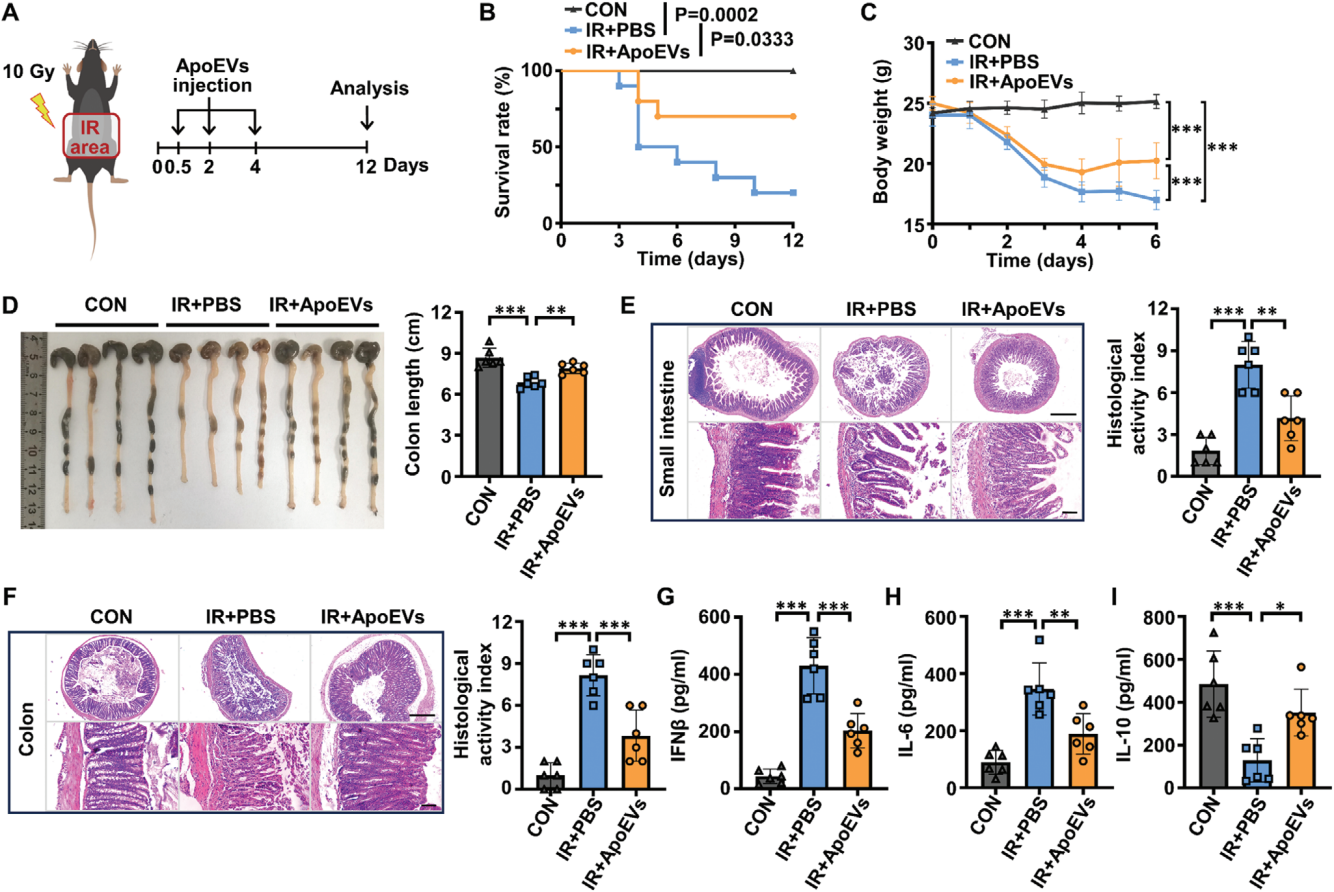
ApoEVs administration alleviates radiation enteritis. A) Therapeutic design for the treatment of radiation enteritis in mice. Mice exposed to ionizing radiation (IR) were intravenously injected with PBS (150 µL) or ApoEVs (150 µg in 150 µL PBS) on days 0.5, 2, and 4 after radiation. B) Survival rate of mice (*n* = 10), significance tested using Log‐rank test. C) Body weight of mice (*n* = 6). D) Representative morphology images of the colon and quantitative analysis of the colon length in each group (*n* = 6). E, F) Representative H&E staining of the E) small intestine and F) colon tissues, and quantitative analysis of the histological activity index (*n* = 6). Scale bar, 500 µm in low‐magnification images and 75 µm in high‐magnification images. G–I) Serum concentrations of G) IFNβ, H) IL‐6, and I) IL‐10 detected by ELISA (*n* = 6). The data are represented as mean ± SD. Statistical analyses are performed by one‐way ANOVA with Tukey's post hoc test. *p < 0.05, **p < 0.01, ***p < 0.001.

As a radiation‐sensitive organ, the intestine is susceptible to injury during abdominal and pelvic radiotherapy, leading to radiation enteritis.^[^ ^1^ ^]^ Recently, we have shown that T cell‐derived ApoEVs have immunoregulatory effects, which promote macrophage transformation toward the anti‐inflammatory phenotype.^[^ ^19^ ^]^ Here, we postulated that ApoEVs contribute to alleviating radiation enteritis. To assess the therapeutic efficacy of ApoEVs in intestinal impairment, ApoEVs were intravenously injected into irradiated mice (**Figure** **2**A). First, edema and erosion were observed in the intestines of mice on the 5th day after irradiation (Figure S2, Supporting Information). In addition, the survival rate and body weight of irradiated mice were significantly decreased in the PBS group (Figure 2B,C). Notably, ApoEVs administration, compared with the PBS group, improved survival and reduced body weight loss in irradiated mice (Figure 2B,C). Moreover, irradiation resulted in a distinct shortening of the colon compared with the control group (Figure 2D). The ApoEVs administration resulted in a significant improvement in colon length compared with the PBS group (Figure 2D). Histological analysis revealed that ApoEVs reduced radiation‐induced inflammatory cell infiltration, crypt damage, and the reduction in the number of villi in the small intestine (Figure 2E) and colon (Figure 2F) tissues.

Cytokines orchestrate the initial immune cell‐driven inflammatory reactivity that may affect the development of enteritis.^[^ ^20^ ^]^ Therefore, we evaluated whether ApoEVs regulated cytokine release. As expected, serum levels of cytokines, such as IFNβ and interleukin‐6 (IL‐6), markedly increased in irradiated mice, and reduced after the treatment of ApoEVs (Figure 2G,H). Simultaneously, the treatment of ApoEVs increased the concentrations of IL­10 compared with the PBS groups (Figure 2I), which may contribute to inflammation resolution and tissue repair. Together, these results strongly indicated that ApoEVs administration improved the survival rate of irradiated mice and ameliorated radiation enteritis.

### 2.3 The Inflammatory Targeting Capacity of ApoEVs

**Figure 3:**
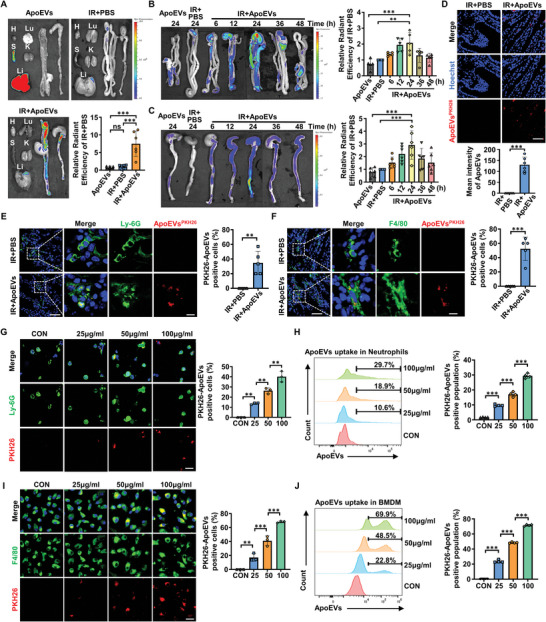
The inflammatory targeting capacity of ApoEVs. A) Representative biodistribution of DiR‐labeled ApoEVs in major organs, small intestine, and colon of mice, and statistical evaluation of intestinal radiant efficiency (*n* = 6). Statistical analysis was performed using the sum of the fluorescence intensities of the small intestine and colon in each group compared with that of the PBS group. H: heart; Li: liver; S: spleen; Lu: lung; K: kidney. B, C) Representative biodistribution of DiR‐labeled ApoEVs at set time points in B) small intestine and C) in colon, and statistical evaluation of small intestine (*n* = 5) and colon (*n* = 6) radiant efficiency. Statistical analysis was performed by comparing the fluorescence intensity of the small intestine or colon in each group with that of the PBS group. D) Confocal microscopy images show the distribution of PKH26‐labeled ApoEVs in the intestines, and the mean fluorescence intensity analysis of ApoEVs in the intestine (*n* = 6). PKH26‐labeled ApoEVs were intravenously injected into irradiated mice. Scale bar, 50 µm. E, F) Confocal microscopy images show the uptake of PKH26‐labeled ApoEVs by E) Ly‐6G‐labeled neutrophils and F) F4/80‐labeled macrophages in vivo. Scale bar, 50 µm in low‐magnification images and 10 µm in high‐magnification images. G) Concentration‐dependent uptake of PKH26‐labeled ApoEVs by mouse bone marrow‐derived neutrophils in vitro. Scale bar, 20 µm. H) Flow cytometric analysis of concentration‐dependent uptake of PKH26‐labeled ApoEVs by neutrophils. I) Concentration‐dependent uptake of PKH26‐labeled ApoEVs by mouse bone marrow‐derived macrophages (BMDM) in vitro. Scale bar, 20 µm. J) Flow cytometric analysis of concentration‐dependent uptake of PKH26‐labeled ApoEVs by BMDM. The data are represented as mean ± SD. Statistical analyses are performed by one‐way ANOVA with Tukey's post hoc test or Student's *t*‐test (two‐tailed) for two‐group comparisons. **p < 0.01, ***p < 0.001; ns, *p* > 0.05.

After clarifying that ApoEVs alleviated radiation enteritis, we then investigated whether ApoEVs had a targeting ability to the intestine. First, DiR‐labeled ApoEVs were injected intravenously into irradiated mice. After injection of ApoEVs into irradiated mice, the fluorescence intensity in the small intestine and colon was stronger than that in the other major organs of this group (**Figure** **3**A(bottom)), demonstrating that ApoEVs could migrate to the intestine. Compared with the PBS group, the ApoEVs group showed a stronger fluorescent signal in the intestine of irradiated mice (Figure 3A), which ruled out the effect of PBS and non‐specific binding of DiR dyes. Moreover, the small intestine and colon in the ApoEVs group after irradiation exhibited a stronger fluorescence intensity compared with the ApoEVs group without irradiation (Figure 3A), indicating that ApoEVs could migrate to the inflammatory region. Next, we analyzed the intestinal targeting of ApoEVs at different time points after injection. We found that ApoEVs distributed in the small intestine and colon at ≈6 h and then reached a peak at 24 h (Figure 3B,C). Subsequently, PKH26‐labeled ApoEVs were injected intravenously into the irradiated mice, and intestinal samples were observed under confocal microscopy, further demonstrating that ApoEVs were recruited to the intestine and internalized by host cells (Figure 3D).

Neutrophils and macrophages could be rapidly recruited to damaged tissues in response to inflammatory signals.^[^ ^2^, ^21^ ^]^ It is well‐accepted that neutrophils and macrophages are professional phagocytic cells, that could internalize apoptotic cells promptly.^[^ ^22^ ^]^ Hence, we speculated that ApoEVs could be taken up by neutrophils and macrophages in the intestine. Undoubtedly, there were apparent red fluorescence signals in the ApoEVs groups compared with the PBS groups (Figure 3E,F), implying the uptake of ApoEVs by Ly‐6G‐labeled neutrophils and F4/80‐labeled macrophages in vivo. We further confirmed the uptake of ApoEVs by neutrophils and macrophages in vitro. First, confocal microscopy results showed that the fluorescence intensity of PKH26‐labeled ApoEVs in neutrophils gradually increased with the elevation of ApoEVs concentration (Figure 3G). In addition, flow cytometric analysis further verified that ≈30% of neutrophils engulfed ApoEVs after incubation with 100 µg mL^−1^ ApoEVs (Figure 3H). Similarly, the phagocytosis of ApoEVs by BMDM was also dependent on the incubation dose of ApoEVs (Figure 3I). Flow cytometry results showed that ≈70% of BMDM phagocytized ApoEVs after incubation with 100 µg mL^−1^ ApoEVs (Figure 3J). Collectively, these data demonstrated that ApoEVs could target inflammatory sites and be taken up by neutrophils and macrophages.

### 2.4 ApoEVs Inhibit the Activation of the cGAS‐STING Pathway

**Figure 4:**
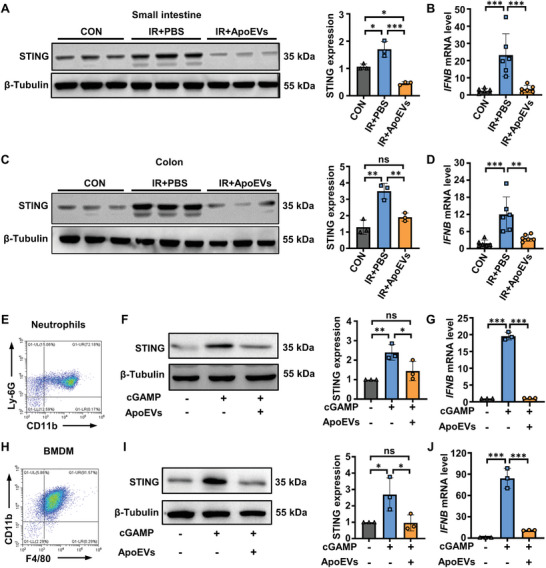
ApoEVs inhibit the activation of the cGAS‐STING pathway. A–D) Irradiated mice were treated with PBS or ApoEVs. Western blot analysis and quantification of STING in the A) small intestine and C) colon. Real‐time PCR was performed to detect mRNA expression of *IFNB* in the B) small intestine and D) colon. E) Representative flow cytometry plots of neutrophils isolated from mouse bone marrow. F, G) ApoEVs were added to cGAMP‐treated neutrophils. The analysis and quantification of F) the STING protein and G) *IFNB* mRNA expression in neutrophils. H) Representative flow cytometry plots of BMDM. I, J) ApoEVs were added to cGAMP‐treated BMDM. The analysis and quantification of the I) STING protein and J) *IFNB* mRNA expression in BMDM. The data are represented as mean ± SD. Statistical analyses are performed by one‐way ANOVA with Tukey's post hoc test. *p < 0.05, **p < 0.01, ***p < 0.001; ns, *p* > 0.05.

DNA double‐strand breaks generated by irradiation are the most lethal form of damage.^[^ ^23^ ^]^ In mammals, the binding of autogenous and allogenic DNA to cGAS allosterically activates cGAS enzymatic activity and leads to the production of cGAMP, a second messenger molecule and powerful agonist of STING.^[^ ^17b^ ^]^ We showed that irradiation enhanced cGAMP production in the small intestine and colon of mice (Figure S3A,C, Supporting Information), and notably activated the expression of STING (Figure S3B,D, Supporting Information). Therefore, inhibiting STING activation is expected to prevent the development of radiation enteritis. Previous studies have revealed that STING activation leads to the production of IFN‐I, causing tissue damage.^[^ ^4^, ^24^ ^]^ We confirmed that the content of IFNβ in the serum of mice increased significantly after irradiation (Figure 2G), which was abrogated by the treatment of ApoEVs. Hence, we speculated that ApoEVs may inhibit the generation of IFNβ by inhibiting STING activation. We found that the STING protein levels in irradiated mice treated with ApoEVs were lower than those in the PBS group (**Figure** **4**A,C). Additionally, we examined the phosphorylation of TBK1 and IRF3 (p‐TBK1 and p‐IRF3), which are key downstream molecules activated by STING and observed a reduction in the expression of p‐TBK1 and p‐IRF3 after ApoEVs treatment. (Figure S4A,B, Supporting Information). Likewise, ApoEVs treatment also suppressed the gene expression levels of *IFNB* in the small intestine and colon (Figure 4B,D).

Given that ApoEVs can be swallowed by neutrophils and macrophages (Figure 3E–J), we further investigated the effect of ApoEVs on STING activation in neutrophils and macrophages. Neutrophils were isolated and treated with cGAMP to activate STING, followed by the addition of ApoEVs to the culture system, with unstimulated neutrophils as a control (Figure 4E–G). Western blot results showed that STING was activated after cGAMP administration, while ApoEVs attenuated the activation of STING (Figure 4F). Similarly, the expression of p‐TBK and p‐IRF3 was decreased after ApoEVs treatment (Figure S4C, Supporting Information). Moreover, the gene expression levels of *IFNB* were significantly reduced in the ApoEVs‐treated group (Figure 4G). Similarly, in the F4/80 and CD11b double positive BMDM, ApoEVs inhibited the STING, p‐TBK, p‐IRF3 and *IFNB* mRNA levels (Figure 4H–J; Figure S4D, Supporting Information). These results indicated that ApoEVs inhibited STING activation induced by irradiation or cGAMP.

To further investigate the mechanism of ApoEVs in inhibiting cGAS‐STING pathway to ameliorate radiation enteritis, we treated irradiated mice with either DMSO, the cGAS inhibitor RU.521, or the STING inhibitor H‐151. We observed that compared with DMSO, RU.521 or H‐151 administration improved survival and body weight loss in irradiated mice (Figure S5A,B, Supporting Information). Additionally, RU.521 or H‐151 treatment led to a significant improvement in colon length compared with the DMSO group (Figure S5C, Supporting Information). Histological analysis further revealed that RU.521 or H‐151 treatment reduced radiation‐induced inflammatory cell infiltration, crypt damage, and the reduction in the number of villi in both small intestine (Figure S5D, Supporting Information) and colon (Figure S5E, Supporting Information) tissues. Simultaneously, to clarify the effectiveness of RU.521 and H‐151 on inhibiting the cGAS‐STING pathway, we detected the phosphorylation of TBK1 and IRF3 and observed that RU.521 or H‐151 administration inhibited the radiation‐induced phosphorylation of TBK1 and IRF3 in mouse colon (Figure S5F, Supporting Information). These findings suggested that inhibiting the cGAS‐STING pathway alleviates radiation enteritis. Moreover, based on the results presented in Figures 2 and 4, we have confirmed that ApoEVs alleviate radiation enteritis and inhibit the cGAS‐STING pathway. Therefore, we concluded that ApoEVs alleviate radiation enteritis by inhibiting the cGAS‐STING pathway.

### 2.5 ApoEVs Hydrolyze cGAMP by ENPP1 to Inhibit the Activation of cGAS‐STING Pathway

**Figure 5:**
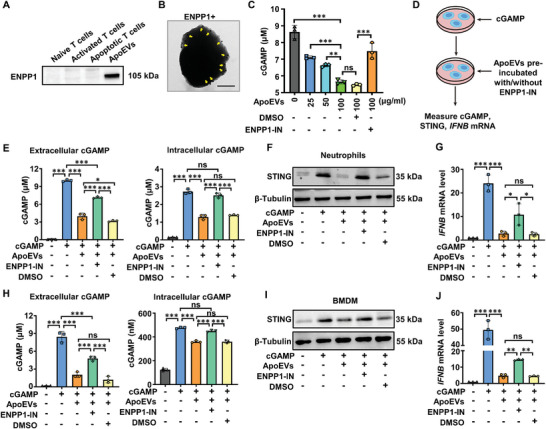
ApoEVs hydrolyze cGAMP by ENPP1 to inhibit the activation of the cGAS‐STING pathway. A) Western blot analysis of ENPP1 expression in naive T cells, activated T cells, apoptotic T cells, and ApoEVs. B) Immunoelectron microscopy detection of ENPP1 on ApoEVs. Scale bar, 200 nm. Arrows indicate ENPP1 adhered by gold particles. C) The activity of ApoEVs to hydrolyze cGAMP with or without ENPP1‐IN (ENPP1 inhibitor, 100 µm) was measured in vitro (*n* = 3). DMSO was used as solvent control. D) Schematic diagram of the experimental procedure. To evaluate the effect of ENPP1 on ApoEVs surface on neutrophils and macrophages, ApoEVs treated with or without ENPP1‐IN were added to cGAMP‐pretreated neutrophils or BMDM. DMSO was used as solvent control. 6 h after ApoEVs were added, the cGAMP concentration, STING protein, and *IFNB* mRNA expression of cells were detected. E) The detection of extracellular and intracellular cGAMP concentration for neutrophils (*n* = 3). F) Western blot analysis of STING protein in neutrophils. G) The mRNA expression of *IFNB* in neutrophils (*n* = 3). H) The detection of extracellular and intracellular cGAMP concentration for BMDM (*n* = 3). I) Western blot analysis of STING protein in BMDM. J) The mRNA expression of *IFNB* in BMDM (*n* = 3). The data are represented as mean ± SD. Statistical analyses are performed by one‐way ANOVA with Tukey's post hoc test. *p < 0.05, **p < 0.01, ***p < 0.001; ns, *p* > 0.05.

We next determined how ApoEVs inhibited STING activation. We found that irradiation enhanced the synthesis of cGAMP, activated STING, and elevated the phosphorylation of TBK1 and IRF3 in the intestines of mice (Figures S3 and S4A,B, Supporting Information). ENPP1 is annotated as an extracellular enzyme that is both a disulfide‐bonded dimerized membrane protein and a secreted protein that hydrolyzes cGAMP.^[^ ^17^, ^25^ ^]^ Since we have confirmed that ApoEVs inhibited the activation of the cGAS‐STING pathway, we wondered whether ApoEVs released by apoptotic T cells could express ENPP1 and hydrolyzed cGAMP. Interestingly, we revealed that ENPP1 was enriched on ApoEVs (**Figure** **5**A). Further determination of ENPP1 localization was performed by immunoelectron microscopy, and the result showed that ENPP1 was located on the membrane surface of ApoEVs (Figure 5B). To further confirm the enzymatic activity of ENPP1 on the surface of ApoEVs, liquid chromatography‐tandem mass spectrometry (LC‐MS/MS) was used to quantify cGAMP. The results suggested that ApoEVs hydrolyze cGAMP dose‐dependently, which can be mitigated significantly by ENPP1‐IN, an inhibitor of ENPP1 (Figure 5C).

Likewise, we wondered whether ENPP1 on the surface of ApoEVs affected the cGAS‐STING pathway of neutrophils and macrophages. To activate cellular STING, we treated neutrophils or BMDM with cGAMP. Then, ApoEVs treated with or without ENPP1‐IN were added to the medium. 6 h later, the cGAMP content, STING expression, and *IFNB* mRNA level of cells were detected (Figure 5D). We observed a dramatic reduction in extracellular and intracellular cGAMP content after ApoEVs were added to neutrophils (Figure 5E). Importantly, this effect was mitigated when ApoEVs were pre‐treated with ENPP1‐IN (Figure 5E). In addition, Western blot results showed that the inhibitory effect of ApoEVs on STING activation and the phosphorylation of TBK1 and IRF was blocked by ENPPI‐IN (Figure 5F; Figure S6A, Supporting Information). Moreover, ApoEVs inhibited *IFNB* mRNA level, and this effect was blocked by ENPPI‐IN (Figure 5G). Similarly, in BMDM, ApoEVs reduced extracellular and intracellular cGAMP and inhibited STING, p‐TBK, p‐IRF3, and *IFNB* mRNA levels while ENPP1‐IN treatment attenuated these effects (Figure 5H–J; Figure S6B, Supporting Information). Given that we have found the inhibitory effect generated by ENPP1 from ApoEVs on the cGAS‐STING pathway, we wonder whether ApoEVs could activate endogenous ENPP1 expression in cells. Therefore, we assessed the transcription level of ENPP1 and found that ApoEVs treatment did not increase *ENPP1* mRNA level in neutrophils or BMDM (Figure S7A,B, Supporting Information). Taken together, these results indicated that the hydrolysis of cGAMP and inhibition of STING activation by ApoEVs were achieved through ENPP1 on their membrane surface.

### 2.6 ApoEVs Hydrolyze Intracellular cGAMP

**Figure 6:**
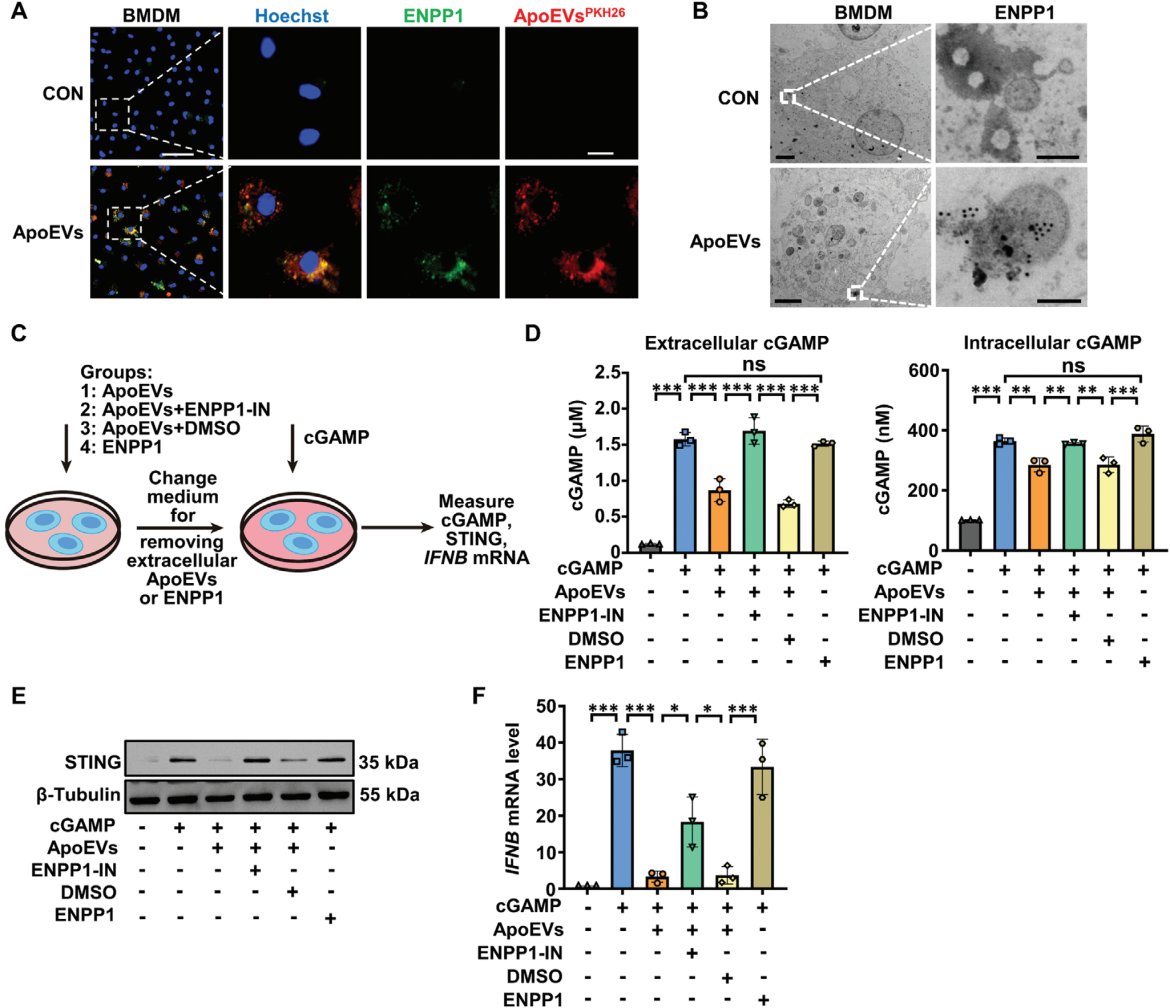
ApoEVs hydrolyze intracellular cGAMP. A, B) The presence of ENPP1 on the surface of intracellular ApoEVs was observed after the uptake of ApoEVs by BMDM. To avoid the effects of ENPP1 on the cell itself, ApoEVs were incubated with ENPP1 antibodies before they were added to BMDM. A) Representative images of PKH26‐labeled ApoEVs (red) and ENPP1 (green) detected by laser scanning confocal microscopy. Scale bar, 50 µm in low‐magnification images and 10 µm in high‐magnification images. B) ENPP1 of intracellular ApoEVs detected by immunoelectron microscopy. Scale bar, 2 µm in low‐magnification images and 200 nm in high‐magnification images. C) Schematic diagram of the experimental procedure. To evaluate the ENPP1 enzymatic activity of intracellular ApoEVs in BMDM, ApoEVs treated with or without ENPP1‐IN (ENPP1 inhibitor, 100 µm) were added to BMDM. DMSO was used as solvent control. After ApoEVs were added, unengulfed ApoEVs were removed by changing the medium, and cells were stimulated with cGAMP for 5 h. Concurrently, by the addition of purified soluble recombinant mouse ENPP1, it was proved that the secretory ENPP1 could not hydrolyze intracellular cGAMP. D) Concentration of extracellular and intracellular cGAMP in BMDM. E) Western blot analysis of the STING expression. F) The mRNA expression of *IFNB*. The data are represented as mean ± SD. Statistical analyses are performed by one‐way ANOVA with Tukey's post hoc test. *p < 0.05, **p < 0.01, ***p < 0.001; ns, *p* > 0.05.

Previous studies have revealed that ENPP1 can degrade extracellular cGAMP, but not intracellular cGAMP.^[^ ^17^, ^25^ ^]^ However, we found that intracellular cGAMP concentrations were reduced after cells were treated with ApoEVs (Figure 5E,H). Moreover, our results showed that macrophages engulfed ApoEVs with high efficiency (Figure 3I,J), thus we wondered whether ENPP1 on the surface of ApoEVs retained enzymatic activity after being phagocytosed by macrophages. First, we determined whether ENPP1 on ApoEVs could be detected in BMDM. Herein, ApoEVs were incubated with ENPP1 antibody and then labeled with PKH26. Then, ApoEVs‐treated BMDM was observed under confocal microscopy. As expected, immunofluorescence staining confirmed that PKH26‐labeled ApoEVs (red) co‐localized with ENPP1 (green) in BMDM (**Figure** **6**A), supporting that ENPP1 on ApoEVs were detected intracellular after BMDM phagocytosed ApoEVs. To further observe the intracellular presence of ENPP1‐containing ApoEVs, BMDM treated with ENPP1 antibody‐incubated ApoEVs was stained with Immunoglobulin G/Gold and detected by TEM. The results showed that these immunogold (black dot) labeled ENPP1‐positive ApoEVs were not encapsulated by endosomes (Figure 6B), which was critical and ensured the contact and reaction between ENPP1 on the surface of ApoEVs and cGAMP.

Subsequently, we examined the intracellular enzyme activity of ENPP1 on the ApoEVs surface. After BMDM phagocytosed untreated ApoEVs or ApoEVs pre‐treated ENPP1‐IN, they were stimulated with cGAMP (Figure 6C). Interestingly, after BMDM phagocytosed ApoEVs, intracellular cGAMP concentration decreased, but this effect was mitigated when ApoEVs were pre‐treated with ENPP1‐IN (Figure 6D). In addition, some studies have suggested the existence of secretory ENPP1 that hydrolyzed only extracellular cGAMP.^[^ ^17^, ^26^ ^]^ To verify that the secretory ENPP1 could not hydrolyze intracellular cGAMP, we set up a group with the addition of purified soluble recombinant mouse ENPP1 when the cells were treated with ApoEVs (Figure 6C). Consistent with previous studies, recombinant mouse ENPP1 treatment failed to reduce the intracellular cGAMP concentration, which indicated that secretory ENPP1 could not hydrolyze intracellular cGAMP (Figure 6D). To further clarify that secretory ENPP1 could not hydrolyze intracellular cGAMP regardless of its concentration, we tested the effect of different concentrations of ENPP1 on intracellular cGAMP content. Our results showed that recombinant ENPP1 could not affect the concentration of intracellular cGAMP (Figure S8, Supporting Information). After BMDM phagocytized ApoEVs, ApoEVs inhibited the expression of STING, p‐TBK, p‐IRF3, and *IFNB* activated by cGAMP (Figure 6E,F; Figure S9, Supporting Information). The above results demonstrated that ENPP1 on ApoEVs retains enzymatic activity after being phagocytosed by cells, which hydrolyzes intracellular cGAMP and inhibits the activation of the cGAS‐STING pathway.

### 2.7 ApoEVs Administration Alleviates Radiation Enteritis by Hydrolyzing cGAMP

**Figure 7:**
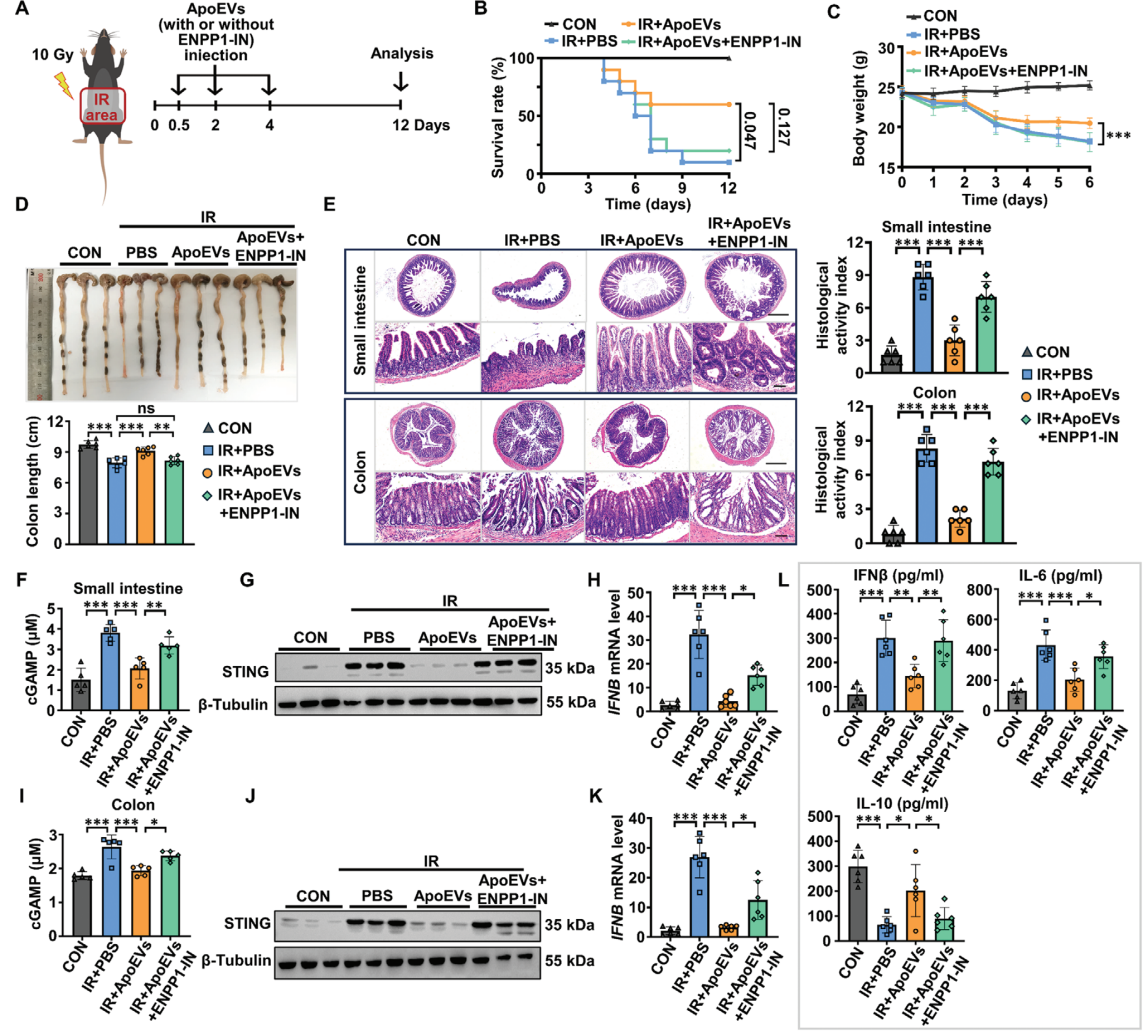
ApoEV administration alleviates radiation enteritis by hydrolyzing cGAMP. A) Schematic diagram of the in vivo experimental procedure. Before ApoEVs injection, ApoEVs were incubated with ENPP1‐IN to block ENPP1 enzyme activity. The mice exposed to ionizing radiation (IR) were intravenously injected with PBS (150 µL), ApoEVs (150 µg in 150 µL PBS), or ENPP1‐IN incubated ApoEVs (150 µg in 150 µL PBS) on days 0.5, 2 and 4 after radiation. B) Survival rate of mice (*n* = 10), significance tested using Log‐rank test. C) Body weight of mice (*n* = 5–6). D) Representative morphology images of the colon and quantitative analysis of the colon length in each group (*n* = 6). E) Representative H&E staining of the small intestine and colon tissues, and quantitative analysis of the histological activity index (*n* = 6). Scale bar, 500 µm in low‐magnification images and 75 µm in high‐magnification images. F–H) cGAMP concentration (*n* = 5, F), STING expression (G), and *IFNB* mRNA level (H) in the small intestine. I–K) cGAMP concentration (*n* = 5) (I), STING expression (J), and *IFNB* mRNA level (K) in the colon. L) Serum concentrations of IFNβ, IL‐6, and IL‐10 detected by ELISA (*n* = 6). The data are represented as mean ± SD. Statistical analyses are performed by one‐way ANOVA with Tukey's post hoc test. *p < 0.05, **p < 0.01, ***p < 0.001; ns, *p* > 0.05.

Previous studies have revealed that cGAMP can be induced by irradiation.^[^ ^25^ ^]^ Since we have demonstrated that ApoEVs hydrolyze extracellular and intracellular cGAMP by ENPP1 in vitro, we wondered whether ApoEVs hydrolyze cGAMP in vivo and improve radiation enteritis in irradiated mice. ApoEVs incubated with or without ENPP1‐IN were intravenously injected into irradiated mice at 12 h, 2 and 4 days after irradiation (**Figure** **7**A). Compared with the ApoEVs group, ApoEVs preincubated with ENPP1‐IN showed more severe intestinal edema and erosion on the 5th day after irradiation (Figure S10A, Supporting Information). While most of the irradiated mice treated with ApoEVs survived, ApoEVs incubated with ENPP1‐IN resulted in a lower survival rate and faster weight loss in irradiated mice (Figure 7B,C). Moreover, the protective effect generated by ApoEVs treatment against colon exfoliation in irradiated mice was eliminated by ENPP‐IN pretreatment (Figure 7D). In addition, compared with ApoEVs treatment, the irradiated mice injected with ENPP1‐IN‐incubated ApoEVs showed more severe inflammatory cell infiltration, crypt damage, and reduction in the numbers of villi in the small intestine and colon tissues (Figure 7E). The above results indicated that ENPP1 on the surface of ApoEVs played an important role in improving radiation enteritis.

To further confirm that the inhibition of the cGAS‐STING pathway by ENPP1 on ApoEVs is required for alleviating radiation enteritis in vivo, we measured cGAMP content, STING protein, and *IFNB* mRNA levels in the small intestine and colon of irradiated mice. We found that, compared with the PBS group, ApoEVs administration decreased cGAMP concentration in the small intestine and colon (Figure 7F,I), whereas ApoEVs incubated with ENPP1‐IN elevated cGAMP concentration (Figure 7F,I). Similarly, ApoEVs inhibited the levels of STING protein (Figure 7G,J), the phosphorylation levels of both TBK1 and IRF3 (Figure S10B,C, Supporting Information), and *IFNB* mRNA (Figure 7H,K) in the intestine of irradiated mice, which could be partially restored by ENPPI‐IN (Figure 7G–K). Thus, these data suggested that ApoEVs hydrolyzed cGAMP through their surface ENPP1, and inhibited the cGAS‐STING pathway in irradiated mice. We further investigated the changes in cytokine levels. Compared with irradiated mice treated with ApoEVs, the serum from irradiated mice that were injected with ENPP1‐IN‐incubated ApoEVs had higher levels of IFNβ, IL‐6, and lower level of IL­10, indicating that ENPP1 on the surface of ApoEVs could downregulate inflammatory response in irradiated mice (Figure 7L). In summary, we concluded that ApoEVs hydrolyzed cGAMP by ENPP1 to regulate the release of cytokines and improve radiation enteritis in irradiated mice.

## 3 Discussion

Radiation became a powerful and effective military, industrial, and medical tool soon after Wilhelm Röntgen's discovery of X‐rays in 1895.^[^ ^27^ ^]^ Simultaneously, reports of radiation‐induced tissue damage have gradually increased, such as the bone marrow, intestine, liver, and lung.^[^ ^28^ ^]^ In addition, as a radiosensitive organ, the intestine is susceptible to injury during pelvic radiotherapy, leading to radiation enteritis with diarrhea and hematochezia.^[^ ^1^ ^]^ Radiation enteropathy is a major issue for long‐term cancer survivors. This progressive condition has few therapeutic options available and can lead to substantial long‐term morbidity and mortality. Here, we found that T cell‐derived ApoEVs treatment prolongs the survival time of irradiated mice and mitigates acute radiation enteritis. It is expected that ApoEVs can be an effective strategy for radiation damage.

Radiation‐induced DNA damage is a key mechanism responsible for radiation cytotoxicity.^[^ ^23^ ^]^ As a cytosolic DNA sensor, cGAS binds to DNA and catalyzes cGAMP synthesis, eventually driving the expression of IFN‐I.^[^ ^17b^ ^]^ Irradiation can activate the cGAS‐STING pathway in the intestine, which triggers acute inflammation.^[^ ^11^, ^29^ ^]^ In addition, activation of the cGAS‐STING pathway is also characteristic of inflammatory bowel disease.^[^ ^7^, ^8^, ^11^ ^]^ Therefore, the cGAS‐STING pathway is an important target for alleviating radiation enteritis and inflammatory bowel disease. Studies have shown that inhibition of cGAS activity or elimination of STING accumulation alleviated colitis.^[^ ^8^, ^9^ ^]^ Previous studies have revealed that STING activation leads to the production of IFN‐I, causing tissue damage.^[^ ^24^ ^]^ However, reducing *IFNB* mRNA levels in the gut may alleviate colitis.^[^ ^30^ ^]^ In addition, IFN‐I release is a key mediator of radiation‐induced liver damage and is described as a mechanism of innate‐immunity‐driven pathology.^[^ ^31^ ^]^ Herein, we found that ApoEVs released by apoptotic T cells can inhibit the cGAS‐STING pathway, which is achieved by ENPP1 on their surface to hydrolyze cGAMP, thus inhibiting innate immunity and improving radiation enteritis.

ENPP1 is located extracellularly, as both a disulfide‐bonded dimerized membrane protein and a secreted protein that is highly abundant in plasma.^[^ ^17^, ^25^ ^]^ In this study, we found that ENPP1 integrated on the ApoEVs surface. Moreover, it has been reported that ENPP1 is the unique cGAMP hydrolase in mammals and degrades only extracellular cGAMP but not intracellular cGAMP.^[^ ^17^, ^25^ ^]^ However, we showed that ApoEVs containing ENPP1 could still be detected and degrade intracellular cGAMP after being phagocytosed by cells. Although we only observed the degradation of intracellular cGAMP by ApoEVs in vitro, we confirmed that ApoEVs can be engulfed by cells in vivo. Therefore, we believe that the degradation of intracellular cGAMP by ApoEVs would also occur in vivo. Previous studies have shown that ENPP1 could not degrade intracellular cGAMP, due to the fact that only secreted ENPP1 and ENPP1 anchored to cell membranes are found.^[^ ^25^, ^32^ ^]^ However, based on the characteristics that extracellular vesicles (EVs) are small and could be phagocytosed by cells, we found that ENPP1 can enter the cell along with ApoEVs and exert its enzymatic activity. Concurrently, we uncovered a new function of EVs the enzyme on the cell surface can be released in the form of EVs and exert its effects within cells.

Apoptosis is an evolutionarily conserved cell death pathway that is responsible for the programmed culling of cells during normal eukaryotic development and maintenance of organismal homeostasis.^[^ ^33^ ^]^ During apoptosis, ApoEVs are released by cells to suppress inflammation and promote cell proliferation and tissue regeneration,^[^ ^19^, ^34^ ^]^ leading to the proposal that the injection of ApoEVs might help to control inflammatory disorder. Our study confirmed that T cell‐derived ApoEVs mitigate radiation enteritis. It has been proven that T cell‐derived EVs are immune suppressive. It has been reported that the enrichment of CD73 on EVs‐derived CD8 T cells hydrolyzes AMP and promotes the production of adenosine, which plays an immunosuppressive role.^[^ ^35^ ^]^ We found that ENPP1 enriched on EV can hydrolyze cGAMP and eliminate inherent immunity. These immunosuppressive molecules on EVs are independent of regulatory T cells, which complement regulatory T cell‐mediated inhibition in inflammatory tissue.^[^ ^35^ ^]^

In the present study, we observed that macrophages exhibit superior efficiency in engulfing ApoEVs, as shown in Figure 3J. On the other hand, we observed a higher hydrolytic efficiency of ApoEVs on intracellular cGAMP in neutrophils, as depicted in Figure 5E,H. We suppose this may be due to the different intracellular fates of ApoEVs. Studies have shown that once extracellular vesicles are bound to recipient cells, they may undergo internalization via various pathways such as clathrin‐mediated or clathrin‐independent endocytosis, as well as endocytosis via caveolae and lipid rafts.^[^ ^36^ ^]^ Upon cellular uptake, extracellular vesicles typically follow the endocytic pathway and accumulate in multivesicular endosomes, which, in most cases, are targeted to lysosomes for degradation.^[^ ^37^ ^]^ In addition, the possibility of extracellular vesicles escaping from endosomes cannot be ruled out, such as endosomal lysis, endosomal permeabilization, and membrane fusion between extracellular vesicles and endosomal membrane.^[^ ^38^ ^]^ Therefore, differences between neutrophils and macrophages in the internalization of ApoEVs, the composition of lysosomes, and the mechanisms controlling the escape of ApoEVs from endosomes may influence the intracellular fates of ApoEVs, leading to the different enzymatic activity of ApoEVs on intracellular cGAMP.

Collectively, our study sheds light on the key role of T cell‐derived ApoEVs in targeting the intestines of abdominal irradiated mice and alleviating radiation enteritis. Mechanistically, the enrichment of ENPP1 on the surface of ApoEVs hydrolyzes intracellular and extracellular cGAMP, thereby inhibiting the cGAS‐STING pathway, promoting inflammation resolution, and alleviating radiation enteritis. This study extends the current understanding of EVs and provides a promising therapeutic strategy for radiation damage.

## 4 Experimental Section

### Mice

Male and female C57BL/6J mice (purchased from Hunan SJA Laboratory Animal Co., Ltd.) aged 6 to 8 weeks were used in this study. The mice were fed with free access to water and food under a 12 h dark/light cycle. All animal experiments were approved by the Institutional Animal Care and Use Committee of the Fourth Military Medical University (2020‐kq‐006).

### Splenic T Cell Culture and Activation

Spleen tissues were dissected from C57BL/6J mice and were gently ground in PBS, followed by centrifugation at 1400 rpm for 7 min. Red cell lysis buffer (Beyotime Biotechnology, C3702) was used to remove red blood cells. Cell suspension was maintained in the RPMI‐1640 medium supplemented with 10% fetal bovine serum (FBS) (Zhejiang Tianhang Biotechnology, 11011–8611). For T cell activation, the plate was coated with anti‐CD3ε antibody (5 µg mL^−1^ in PBS) (BioLegend, 100340) for 2 h at 37 °C prior to cell seeding, and then the cells were cultured in RPMI 1640 medium with anti‐CD28 antibody (2 µg mL^−1^ in PBS) (BioLegend, 102116). After 48 h of incubation, T cells were activated. Activated T cells were determined by flow cytometry (Beckman Coulter, USA) using allophycocyanin (APC)‐conjugated anti‐CD25 antibody (BioLegend, 102012).^[^ ^18^ ^]^

### Isolation and Characterization of ApoEVs

Activated T cells were treated with staurosporine (Cell Signaling Technology, 9953) at 500 nm for 12 h to induce apoptosis. Then, cells and culture media were collected and centrifuged at 800 g for 10 min to remove the cells and debris. The supernatant was transferred to a new tube and centrifuged at 16 000 g for 30 min to concentrate ApoEVs. The supernatant was removed, and the pellet was resuspended in PBS for subsequent experiments. The concentration of ApoEVs was quantified using the BCA protein assay kit (TIANGEN, PA115).^[^ ^15c^ ^]^

The morphology of ApoEVs was observed by scanning electron microscope (SEM) (Hitachi) and transmission electron microscope (TEM) (Thermo Fisher), with a PHURONA camera (EMSIS) and RADIUS 2.0 software (EMSIS). The size distribution of ApoEVs was detected using the Litesizer 500 (Anton Paar). For phosphatidylserine (PtdSer) detection, ApoEVs were stained with PE‐Annexin V (BD Biosciences Pharmingen, 559763). For detecting the surface markers, ApoEVs were stained with anti‐mouse C1q antibodies (Abcam, ab71940), followed by fluorescence images captured with CLSM (Nikon). For apoptotic marker detection, ApoEVs were characterized by Western blot using anti‐Caspase‐3 antibodies.^[^ ^15c^ ^]^

### Animal Models of Abdominal Irradiation and Treatment

Male C57BL/6J mice aged 6 to 8 weeks were anesthetized and their abdomens (3 cm wide, from the xiphoid process of the sternum to the symphysis pubis) were exposed to X‐ray irradiation with a total dose of 10 Gy at a rate of 1.3 Gy min^−1^. The rest of the body was protected with a 2 cm thick lead against irradiation. Irradiated mice were randomly grouped and intravenously injected with PBS (150 µL) or ApoEVs (150 µg in 150 µL PBS) on days 0.5, 2, and 4 after irradiation. In addition, to generate cGAS‐ or STING‐inhibited irradiated mice, irradiated mice were divided into three groups randomly and received intraperitoneal injections daily for 7 days with DMSO, cGAS inhibitor RU.521 (500 µmol, in 200 µL corn oil 10% DMSO, MedChemExpress), or STING inhibitor H‐151 (750 nmol, in 200 µL PBS 5% Tween‐80, MedChemExpress).^[^ ^39^ ^]^ The body weight of mice was recorded, and their survival status was monitored daily.

### Histological Analysis

Small intestine and colon tissues were fixed in 4% paraformaldehyde (PFA) overnight at 4 °C before being processed and embedded in paraffin. 4‐µm thick sections were stained by H&E in an automated stainer (Leica Autostainer XL). Images were obtained with an Olympus BX41 Microscope (Olympus). The histological score was graded according to the following criteria: i) Inflammatory cell infiltrate was evaluated by the number of leukocyte foci: 0‐ no significant change, 1‐ mild, infiltrated leukocytes in focal or occasional, 2‐ moderate, infiltrated leukocytes with more than one focus and 3‐ severe, infiltrated leukocytes diffuse or continuous; ii) Erosion was evaluated by the loss of surface epithelium: 0‐ no erosion, 1‐ one focus, 2‐ multiple foci and 3‐ continuous surface loss; iii) Crypt abscesses were evaluated by the neutrophils in crypt lumen: 0‐ no crypt abscess, 1‐ rare crypt abscesses, 2‐ multiple crypt abscesses and 3‐ crypt lost, surface epithelium present, 4‐ crypt and surface epithelium lost. The histological score was the sum of the above three parameters.

### Isolation and Characterization of Mouse Bone Marrow‐Derived Macrophages (BMDM)

Primary macrophages were obtained by collecting bone marrow from femurs and tibias of C57BL/6J wild‐type mice. Bone marrow cells were flushed out with PBS and lysed with red cell lysis buffer. Bone marrow cells were collected and were cultured in DMEM (high glucose) supplemented with 10% FBS, 1% penicillin/streptomycin, and 20 ng mL^−1^ macrophage colony‐stimulating factor (M‐CSF) (PeproTech, 315‐02) for 7 days. After induction for 7 days, cells were used in subsequent experiments. The adherent cells were differentiated macrophages, which were confirmed by flow cytometry with FITC‐conjugated anti‐mouse F4/80 antibodies (BioLegend, 123110) and PerCP/Cy5.5‐conjugated anti‐CD11b antibodies (BioLegend, 101227).

### Isolation and Characterization of Mouse Bone Marrow‐Derived Neutrophils

Neutrophils were isolated from the bone marrow of C57BL/6J wild‐type mice using Histopaque separation media by a density gradient centrifugation. In brief, bone marrow cells were harvested from the femurs and tibias, followed by erythrocyte lysis with red cell lysis buffer. Then, the cell suspension was overlaid on top of the Histopaque 1077 (Sigma, 10771) layer with Histopaque 1119 (Sigma, 11191) at the bottom. After centrifugation, neutrophils at the interface of the Histopaque 1119 and Histopaque 1077 layers were collected. Then, the neutrophils were cultured in RPMI 1640 medium supplemented with 10% FBS and 1% penicillin/streptomycin. Neutrophil purity was determined by flow cytometry using allophycocyanin (APC)‐conjugated anti‐CD11b (BioLegend, 101212) and phycoerythrin (PE)‐conjugated anti‐Ly‐6G (BioLegend, 127607) antibodies.

### In Vivo Biodistribution of ApoEVs

ApoEVs were prelabeled with DiR (Yeasen Biotechnology, 40757ES25) according to the manufacturer's instructions, and the labeled ApoEVs were intravenously injected into the irradiated mice (150 µg per mouse). At the set time points, irradiated mice were euthanized, and small intestines, colons, and major organs were collected for imaging using an in vivo imaging system (IVIS).

### Uptake of ApoEVs by Macrophages and Neutrophils

To detect the uptake of ApoEVs by BMDM and neutrophils in vitro, ApoEVs were pre‐labeled with PKH26 (Sigma–Aldrich, MINI26) according to the manufacturer's instructions. PKH26‐labeled ApoEVs were added to the culture system at indicated concentrations.

To detect the uptake of ApoEVs by macrophages and neutrophils in vivo, PKH26‐labeled ApoEVs (150 µg per mouse) were injected into the irradiated mice via the tail vein, with PBS as a control. After 24 h, the mice were euthanized, and the small intestine and colon were obtained and fixed with 4% paraformaldehyde (PFA) overnight at 4 °C.

### Immunofluorescence Staining

Cells and intestinal tissues were fixed with 4% PFA. Specifically, intestinal tissues were dehydrated with 30% sucrose solution at 4 °C overnight, embedded in a Frozen Section Compound (Leica), and cut into 10‐µm cryosections. Cells or intestinal sections were incubated in the blocking buffer for 30 mins before incubation with primary antibodies ENPP1 (HUABIO, ER1908‐13), Ly‐6G (Santa Cruz Biotechnology, sc‐53515), or F4/80 (Abcam, ab6640) at 4 °C overnight. After washing, samples were stained with FITC‐conjugated IgG secondary antibody for 2 h at room temperature. Cell nuclei were counter‐stained with Hoechst 33342 (Sigma–Aldrich, 14533) for 10 min at room temperature. Fluorescence images were captured by CLSM.

### Protein isolation and Western blot analysis

Protein samples were extracted using RIPA buffer (150 mm NaCl, 1% Triton X‐100, 0.5% sodium deoxycholate, 0.1% SDS, 50 mm Tris pH 8.0) supplemented with a protease inhibitor cocktail tablet (Sigma–Aldrich, s8830). Total protein concentration was assessed with the BCA protein assay kit. Protein samples (20 µg) were loaded and separated by SDS‐polyacrylamide gel electrophoresis (PAGE) and transferred onto polyvinylidene fluoride (PVDF) membranes (Millipore, 03010040001). The membranes were blocked in 5% bovine serum albumin (BSA) for 2 h at room temperature and then incubated with primary antibodies overnight at 4 °C. After washing three times with PBST (PBS containing 0.1% Tween 20), the membranes were incubated with secondary antibodies for 2 h at room temperature. Protein bands were visualized with a Western‐Light Chemiluminescent Detection System (Tanon). Quantification of digital images was performed using ImageJ software. The following primary antibodies were used: Caspase‐3 (Cell Signaling Technology, 9665), CD11b (Abcam, ab133357), CD44 (Abcam, ab189524), CD3 (Santa Cruz Biotechnology, sc‐20047), STING (Abcam, ab288157), ENPP1 (Abcam, ab217368), GAPDH (CWBIO, CW0100), phospho‐TBK1 (Ser172) (ABclonal, A2573), phospho‐IRF3 (Ser396) (Proteintech, 29528‐1‐AP) and β‐tubulin (CWBIO, CW0098),.

### Electron Microscopy

For TEM, a drop of suspension containing ApoEVs was applied to a 200‐mesh carbon‐stabilized copper grid. ApoEVs were allowed to absorb for 5 min before the excess suspension was wicked off. Next, the grid was stained with phosphotungstic acid hydrate (pH 6.5) for 30 s followed by washing with distilled water three times. Excess solution was wicked off and the grid was allowed to air‐dry before observation.

For immunogold staining, ApoEVs were incubated with the primary antibodies ENPP1 (1:200) (HUABIO, ER1908‐13) overnight at 4 °C. Then, ApoEVs were applied to 200‐mesh nickel grids. After blocking with 5% BSA, the grid was incubated with the Rabbit Immunoglobulin G/Gold (1:50) (Bioss Antibodies, bs‐0295P‐Gold) for 30 min. The grid was then washed with ultrapure water and 2.5% glutaraldehyde, and stained with phosphotungstic acid hydrate (pH 6.5) for 30 s, followed by rinsing with ultrapure water. After dying, all grids were examined by TEM (TECNAI Spirit, FEI).^[^ ^40^ ^]^

### ELISA Assay

For serum examination, mice were anesthetized and the whole peripheral blood was obtained. After that, serum was isolated by centrifuging at 4000 rpm for 20 min. The serum was diluted fivefold with the sample diluent. The concentrations of IL‐10 (NeoBioscience, EMC005), IL‐6 (NeoBioscience, EMC004), and IFNβ (Bioss Antibodies, bsk12073) in serum were detected using murine ELISA kits following the manufacturer's instructions.

### RNA Extraction and Quantitative Real‐Time PCR

Total RNA was extracted from cells and tissue with TRIzol reagent (Mishu Shengwu, MI00617) and quantified by Nanodrop (Thermo Fisher Scientific). Then, 1 µg of total RNA was converted into cDNA with the Reverse Transcription Kit (TaKaRa, RR037A). mRNA levels were quantified using the SYBR Green Master Mix (TaKaRa, RR820B) and detected by CFX 96Touch (Bio‐Rad). Relative expression of genes was calculated by the −2ΔΔCt method. GAPDH was used as a housekeeping gene. Primers used in this study are listed below: IFNβ (Forward: 5′‐CCTGGAGCAGCTGAATGGAA‐3′; Reverse: 5′‐CCACCCAGTGCTGGAGAAAT‐3′), GAPDH (Forward: 5′‐TGTGTCCGTCGTGGATCTGA‐3′; Reverse: 5′‐TTGCTGTTGAAGTCGCAGGAG‐3′), and ENPP1 (Forward: 5′‐GAGAGGAGCCGCTGGAGAAG‐3′; Reverse: 5′‐ACACATACTGACAAAACCAGCG‐3′).

### Measurement of the cGAMP Hydrolytic Activity of ApoEVs

In vitro, to detect the cGAMP hydrolytic activity of ApoEVs, ApoEVs were repelleted by centrifuged at 16 000 g for 30 min and resuspended in DMEM (free EVs). Subsequently, 0, 25, 50, 100 µg mL^−1^ ApoEVs added with or without 100 µm ENPP1 inhibitor (ENPP1‐IN‐12, MedChemExpress, HY‐143256) were reacted with 10 µm cGAMP (MedChemExpress, HY‐100564A) in 100 µL of DMEM (free EVs) for 2 h at room temperature. For cell measurements, extracellular cGAMP was measured directly from cell supernatants. To quantify intracellular cGAMP, cells were washed once with PBS and then lysed in 100 µL RIPA buffer for 15 min on ice. Lysates were then cleared of insoluble material before being used for cGAMP measurement. To quantify cGAMP in mouse intestinal tissues, intestines were cleaned and weighed, minced, and then lysed in 300 µL RIPA buffer for 15 min on ice. Lysates were then cleared of insoluble material before being used for cGAMP measurement. All samples were diluted five times with dilution buffer (Tris HCl pH 7.7 100 mm, NaCl 150 mm, CaCl_2_ 2 mm, ZnCl_2_ 200 µm). The diluted samples were immediately loaded to LC‐MS/MS for cGAMP quantification. The LC‐MS/MS analysis was performed on an AB Sciex X500R Triple Quad mass spectrometer with the electrospray ionization source. Compound cGAMP (MedChemExpress, HY‐100564A) was used as a standard sample.

### Statistical Analysis

Data are presented as means ± SD of at least triplicate measurements and were analyzed using SPSS v.19.0 software. Statistical analysis was performed by Student's *t*‐test (two­tailed), or one‐way ANOVA. Tukey's post hoc test was used for multiple post hoc comparisons to determine the significance between the groups after one‐way ANOVA. Survival curves were compared using the log‐rank test. The difference between groups was considered statistically significant for *p < 0.05, very significant for **p < 0.01, and the most significant for ***p < 0.001. Graph analysis was performed using GraphPad Prism 9.

## Conflict of Interest

The authors declare no conflict of interest.

## Author Contributions

Y.Z., L.B., and S.G. contributed equally to this work. Y.Z., L.B., and S.G. contributed to the experimental performing, data acquisition and analysis, and manuscript drafting; G.D. and Z.L. contributed to animal experiments; F.D. and H.L. contributed to data analysis and interpretation; Z.W. and L.Y. contributed to flow cytometry analysis; X.L. contributed to data interpretation; Sh.L., X.Y., and Si.L. contributed to the study conception and design, data interpretation, and manuscript revision. All authors read and approved the final version of the manuscript.

## Supporting information

Supporting Information

## Data Availability

The data that support the findings of this study are available from the corresponding author upon reasonable request.;
