# Supplementary material for: T Cell‐Derived Apoptotic Extracellular Vesicles Hydrolyze cGAMP to Alleviate Radiation Enteritis via Surface Enzyme ENPP1
Source: Adv Sci (Weinh). 2024 Jun 18;11(31):2401634. doi: 10.1002/advs.202401634 (PMC11336903; doi:10.1002/advs.202401634)
Supplement: Supplementary file 1 — Supporting Information [file ADVS-11-2401634-s001.pdf]

ADVANCED SCIENCE
Open Access

# Supporting Information

for *Adv. Sci.*, DOI 10.1002/advs.202401634

T Cell-Derived Apoptotic Extracellular Vesicles Hydrolyze cGAMP to Alleviate Radiation Enteritis via Surface Enzyme ENPP1

*Yang Zhou, Lili Bao, Shengkai Gong, Geng Dou, Zihan Li, Zhengyan Wang, Lu Yu, Feng Ding, Huan Liu, Xiayun Li, Siying Liu*, Xiaoshan Yang* and Shiyu Liu**

## Supplemental Figures

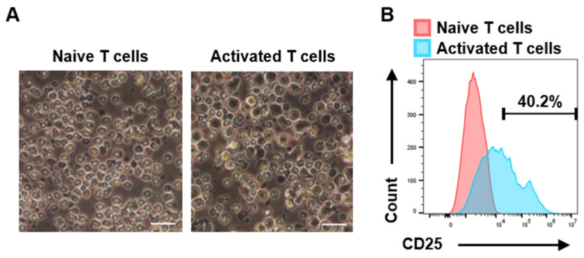


**Figure S1. Characterization of naive and activated T cells.** A) Representative photographs of naive and activated T cells. Scale bar, 500 μm. B) Flow cytometry analysis of CD25 expression in naive and activated T cells.

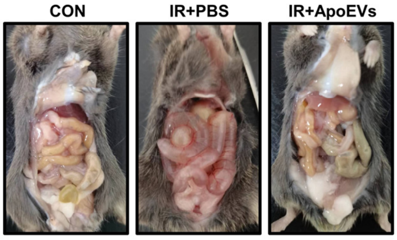


**Figure S2. ApoEVs administration alleviates radiation enteritis.** The degree of intestinal edema and erosion was observed in the irradiated mice on the 5th day after irradiation.

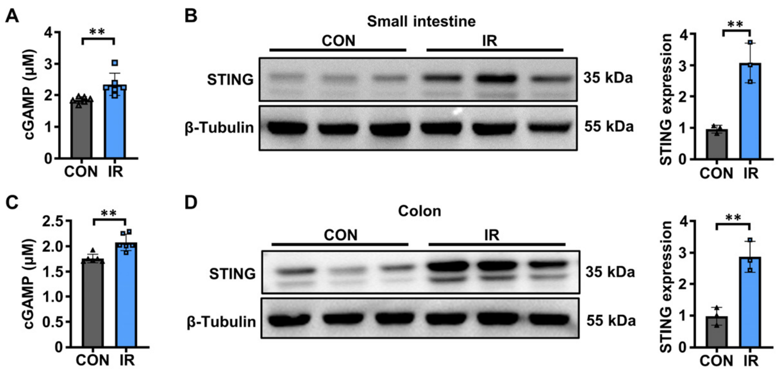


**Figure S3. Irradiation activates the cGAS-STING pathway in the small intestine and colon of mice.** A) Concentration of cGAMP in the small intestine. B) STING expression in the small intestine. C) Concentration of cGAMP in the colon. D) STING expression in the colon. The data are represented as mean ± SD. Statistical analyses are performed by Student's t test (two-tailed) for two group comparisons. **p < 0.01.

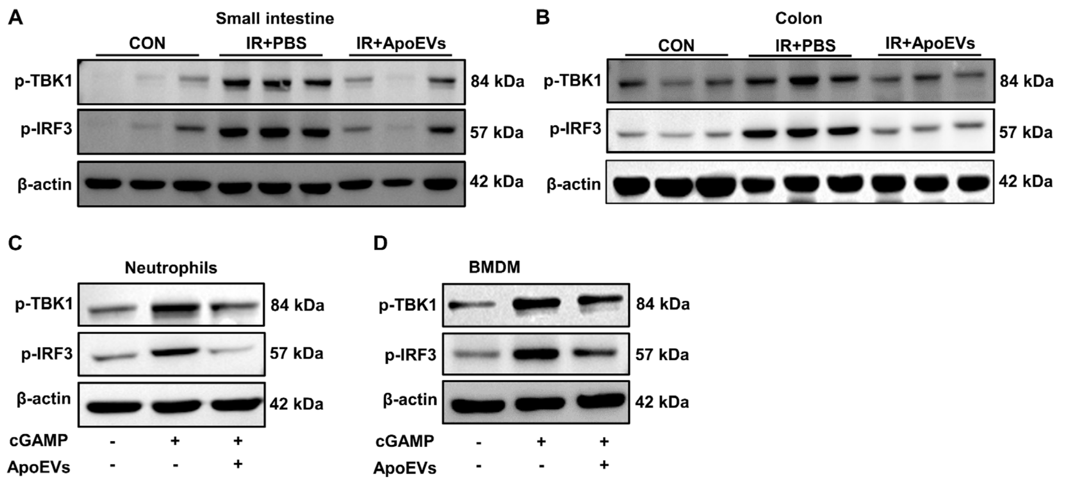


**Figure S4. ApoEVs inhibited the phosphorylation of TBK1 and IRF3 induced by irradiation or cGAMP.** A,B) Western blot analysis of p-TBK1 and p-IRF3 expression in the small intestine (A) and colon (B). C) ApoEVs were added to cGAMP-treated neutrophils. Western blot analysis of p-TBK1 and p-IRF3 in neutrophils *in vitro*. D) ApoEVs were added to cGAMP-treated BMDM. Western blot analysis of p-TBK1 and p-IRF3 in BMDM *in vitro*.

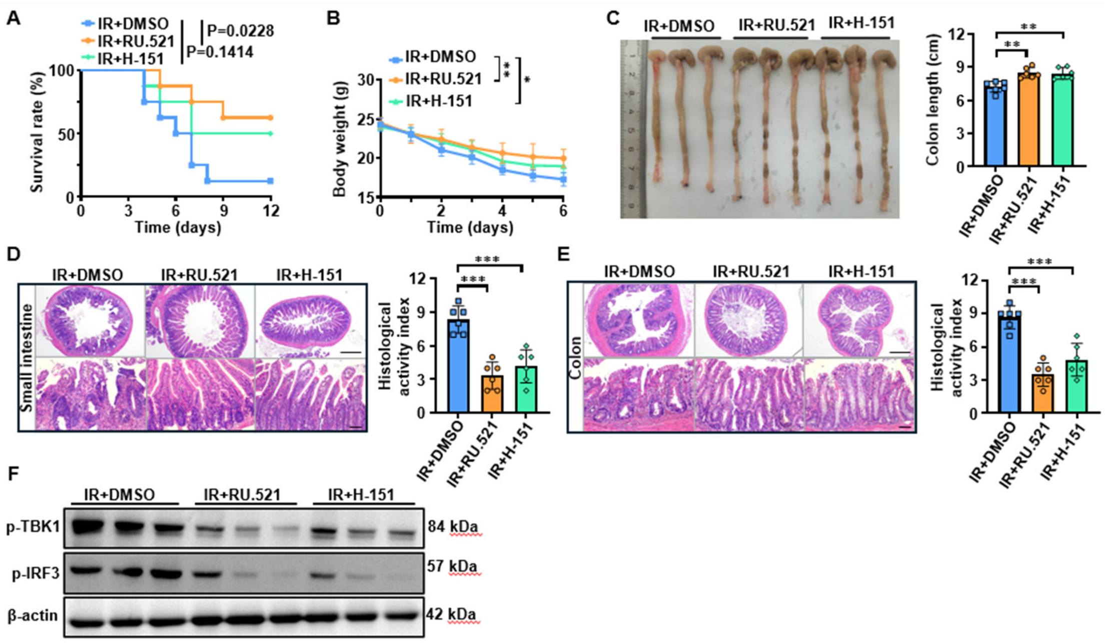


**Figure S5. Inhibition of cGAS-STING pathway alleviated radiation enteritis.** A) Survival rate of mice (n = 8), significance tested using Log-rank test. B) Body weight of mice (n = 6). C) Representative morphology images of the colon and quantitative analyses of the colon length in each group (n = 6). D,E) Representative H&E staining of the small intestine (D) and colon (E) tissues, and quantitative analysis of the histological activity index (n = 6). Scale bar, 500 μm in low-magnification images and 75 μm in high-magnification images. F) Western blot analysis of p-TBK1 and p-IRF3 expression in the colon. The data are represented as mean ± SD. Statistical analyses are performed by one-way ANOVA with Tukey's post hoc test. **p < 0.01, ***p < 0.001.

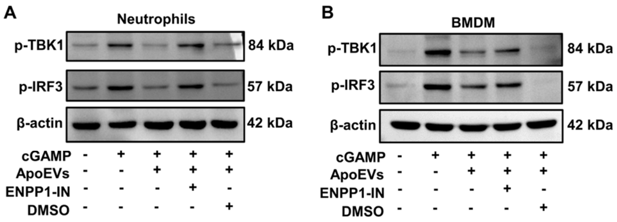


**Figure S6. ApoEVs inhibit the phosphorylation of TBK1 and IRF3 via surface ENPP1.** A) Western blot analysis of p-TBK1 and p-IRF3 in neutrophils. B) Western blot analysis of p-TBK1 and p-IRF3 in neutrophils.

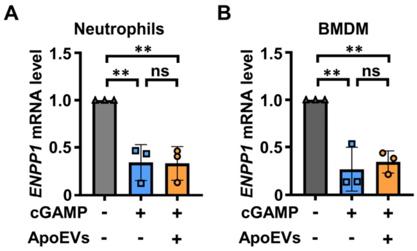


**Figure S7. The analysis of the *ENPP1* mRNA expression in neutrophils and macrophages.** A) The analysis of the *ENPP1* mRNA expression in neutrophils. ApoEVs were added to cGAMP-treated neutrophils. B) The analysis of the *ENPP1* mRNA expression in BMDM. ApoEVs were added to cGAMP-treated BMDM. The data are represented as mean ± SD. Statistical analyses are performed by one-way ANOVA with Tukey's post hoc test for multiple group comparisons. one-way ANOVA with Tukey's post hoc test. **p < 0.01; ns, p > 0.05.

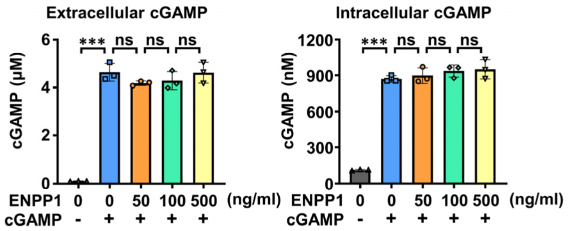


**Figure S8. The secretory ENPP1 cannot enter the cell or hydrolyze intracellular cGAMP.** The detection of extracellular and intracellular cGAMP concentration. To verify that the secretory ENPP1 cannot enter the cell and hydrolyze cGAMP, different concentrations of recombinant mouse ENPP1 were added to cGAMP-treated BMDM. Five hours after ENPP1 was added, unengulfed ENPP1 was removed by changing the medium, and cells were stimulated with cGAMP for 5 h. The data are represented as mean ± SD. Statistical analyses are performed by one-way ANOVA with Tukey's post hoc test. ***$p < 0.001$; ns, $p > 0.05$.

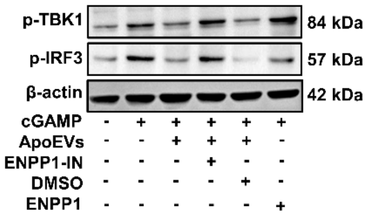


**Figure S9. ApoEVs hydrolyze intracellular cGAMP by ENPP1 and inhibit the phosphorylation of TBK1 and IRF3.** Western blot analysis of p-TBK1 and p-IRF3 in BMDM.

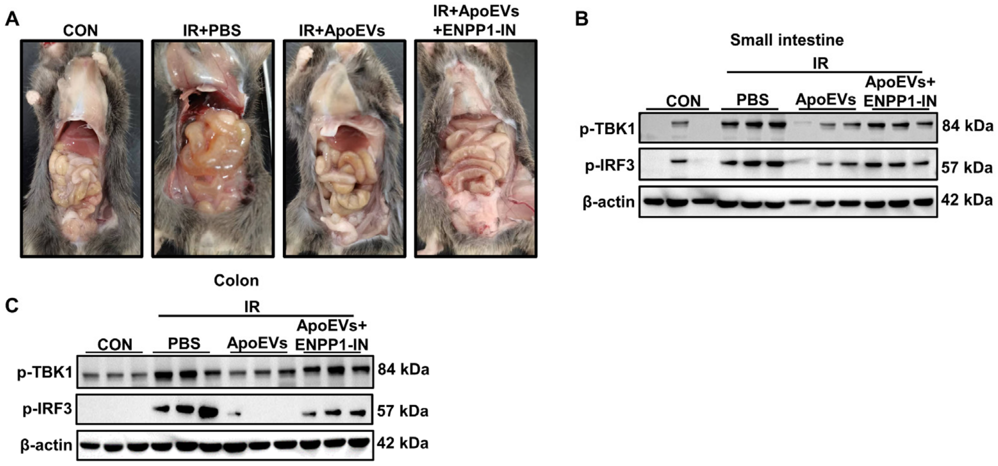


**Figure S10. ApoEVs administration alleviates radiation enteritis by ENPP1.** A) The degree of intestinal edema and erosion was observed in the irradiated mice on the 5th day after irradiation. B,C) Western blot analysis of p-TBK1 and p-IRF3 expression in the small intestine (B) and colon (C).
